# Effect of Bednets and Indoor Residual Spraying on Spatio-Temporal Clustering of Malaria in a Village in South Ethiopia: A Longitudinal Study

**DOI:** 10.1371/journal.pone.0047354

**Published:** 2012-10-12

**Authors:** Eskindir Loha, Torleif Markussen Lunde, Bernt Lindtjørn

**Affiliations:** 1 School of Public and Environmental Health, Hawassa University, Hawassa, Ethiopia; 2 Centre for International Health, University of Bergen, Bergen, Norway; 3 Geophysical Institute, University of Bergen, Bergen, Norway; Kenya Medical Research Institute (KEMRI), Kenya

## Abstract

**Background:**

Understanding the spatio-temporal pattern of malaria transmission where prevention and control measures are in place will help to fine-tune strategies. The objective of this study was to assess the effect of mass distribution of bednets and indoor residual spraying (IRS) with insecticides on the spatio-temporal clustering of malaria in one malaria endemic village in south Ethiopia.

**Methods:**

A longitudinal study was conducted from April 2009 to April 2011. The average population was 6631 in 1346 locations. We used active and passive searches for malaria cases for 101 weeks. SatScan v9.1.1 was used to identify statistically significant retrospective space–time clusters. A discrete Poisson based model was applied with the aim of identifying areas with high rates. PASW Statistics 18 was used to build generalized Poisson loglinear model.

**Results:**

The total number of both types of malaria episodes was 622, giving 45.1 episodes per 1000 persons per year; among these, episodes of *Plasmodium falciparum* and *vivax* infection numbered 316 (22.9 per 1000 per year) and 306 (22.2 per 1000 per year), respectively. IRS with Dichlorodiphenyltrichloroethane (DDT) and later with Deltamethrin and free mass distribution of insecticide-treated nets (ITNs) were carried out during the study period. There was space**–**time clustering of malaria episodes at a household level. The spatio-temporal clustering of malaria was not influenced by free mass distribution of ITNs; however, the time-span of the spatio-temporal clustering of malaria cases ended after IRS with Deltamethrin. The presence of clusters on the south-east edge of the village was consistent with the finding of an increasing risk of acquiring malaria infection for individuals who lived closer to the identified vector breeding site.

**Conclusion:**

The risk of getting malaria infection varied significantly within one village. Free mass distribution of ITNs did not influence the spatio-temporal clustering of malaria, but IRS might have eliminated malaria clustering.

## Introduction

Malaria is a leading health problem in Ethiopia, where 67% of the 82 million people are estimated to be at risk. There were 1 036 316 confirmed cases of malaria in 2009. The dominant plasmodium species are *Plasmodium falciparum* and v*ivax,* and the major *Anopheles* species responsible for transmission is *arabiensis* [1]. In 2005, massive expansion of malaria control programmes included the distribution of long-lasting insecticidal nets (LLINs), and the use of Artemether–Lumefantrine as a first-line treatment for *Plasmodium falciparum* malaria [1], [2]. In 2007 and 2010, the percentage of households with at least one LLTN was 53.8% [2] and 72% [1], respectively. In addition, 20% of households below an altitude of 2000 metres above sea level were subjected to indoor residual spraying (IRS) [2].

Nationally, the number of cases has declined since 2005 due to an expansion in the malaria control programmes. However, malaria admissions increased in 2009 [1], and more than the expected number of cases occurred in south Ethiopia in 2010/2011 [3]. The reasons for this apparent increase are not well documented and there have been calls for better understanding of disease transmission, and an evaluation of malaria control measures.

To implement malaria prevention and control measures, and to understand risk dynamics, application of Geographic Information System (GIS) has been emphasized, in particular to provide a precise definition of the time and location of epidemics [4]. The results of GIS can be used to explain the interactions among humans, their environment, risk factors, and changes over time and space [5], [6]. Geo-referencing of malaria cases, combined with efforts to link them to potential locations of environmental exposure, increases the benefit of disease maps [7].

Spatial epidemiological studies at a finer geographic scale, such as households, help to increase understanding of the varied pattern of malaria infection and transmission [8]. Several studies have reported significant spatial and temporal variation in malaria transmission. Mapping high-risk zones or clusters may contribute to improved prevention and control efforts by delivering limited resources to the population at higher risk [8]–[14]. To inform policy, it is also important to observe the spatio-temporal pattern of transmission in line with the presence or absence of prevention and control measures on the ground. Therefore, this study aimed to investigate the effect of prevention and control measures on the spatio-temporal clustering of malaria at a household level in one malaria endemic village in south Ethiopia.

## Materials and Methods

### Study Area

**Figure 1:**
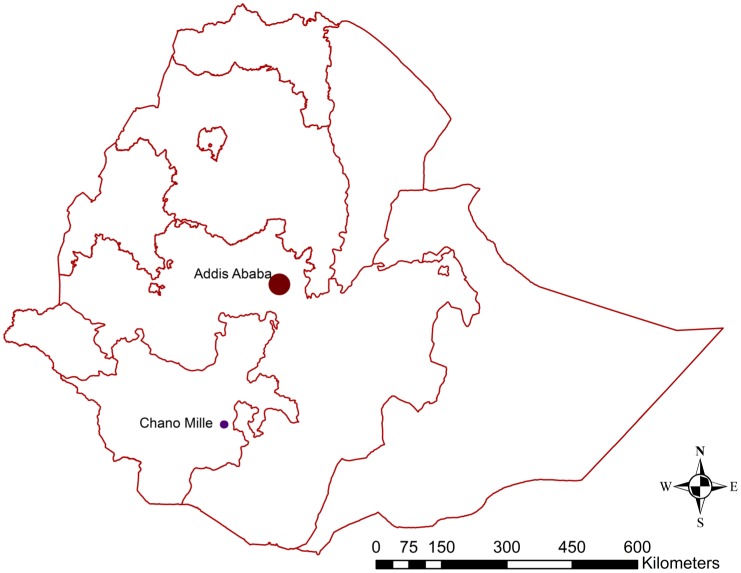
Map of Ethiopia and location of Chano Mille Kebele.

Chano Mille Kebele (Kebele is the lowest administrative unit in Ethiopia) is 492 km south of Addis Ababa (Figure 1). The altitude is 1206 m above sea level. The annual rainfall was 650 mm in 2009 and 1057 mm in 2010. The area covers 2.4 square km. There were 1388 households (1346 locations) with an average population (from the first, midway and last census) of 6631 people (the total population followed was 8121). The average household size was 5.9 (8121/1388) individuals. The number of households was greater than the number of locations because some households left the study area, replaced by others in the same locations.

Chano Mille Kebele was selected purposely for the study of malaria epidemiology. Three main irrigation ditches run from the neighbouring Kebele in the west, cross the Kebele and may also end within the Kebele. There are two adjacent Kebeles, Chano Dorga to the north-west and Chano Chalba to the south-west. The area to the east and south-east sides of the Kebele, extending to Lake Abaya, is used for agricultural purpose. Most of the households grow mango trees within their compounds.

There was one health post in the village staffed by a health extension worker. A health post provides basic health services, including malaria diagnosis using rapid diagnostic test (RDT) kits and treatment with Artemether–Lumefantrine.

### Study Design

The cohort study was carried out from April 2009 to April 2011. Both active and passive surveillance schemes were used. Each household was visited every week for 101 weeks looking for cases of fever (temperature ≥37.5 degrees Celsius); if needed patients were referred to the health post for diagnosis and treatment of malaria (active case finding). Each day, we checked whether the referred cases had visited the health post. During the days between the visits, the residents were advised to self-report to the health post if they became febrile (passive case-finding).

We gave a unique household number to each household before the first census. The geographic coordinates of all households were recorded using GPS during the first census. The GPS reading for the new households was performed during the midway census. We also recorded GPS coordinates for the main vector breeding sites. These vector breeding sites are swampy areas close to Lake Abaya, with many hoof prints of cattle and hippopotami. Such small water bodies are formed mainly after flooding of the lake during the rainy season. We did not find larvae of *Anopheles* species near the irrigation ditches or at other locations in the study area.

### Blood Samples and Patient Management

The laboratory technician used a single finger prick to collect blood samples for RDT and prepared thick and thin blood films for microscopic evaluation. Based on the results of the RDT, the patients were treated with Artemether–Lumefantrine (*Plasmodium falciparum*) or Chloroquine (*Plasmodium vivax*). Chloroquine was provided by the research project. Two experienced laboratory technicians read each blood slide independently. Whenever there was a discordant reading, confirmation was obtained from a third reader. All readers were blinded to the readings of the others.

### Analysis

#### Spatio-temporal

We used SatScan v9.1.1 (http://www.satscan.org/) software for spatial and space–time statistical analysis, to identify statistically significant retrospective space–time clusters. Two episodes of malaria (one vivax and one falciparum) were excluded from the analysis because of missing data on the location. The time precision was 1 month and a coordinate file was provided with latitude and longitude values. All cases were checked to ascertain whether they had occurred within the specified time period and geographical area. Case, population and coordinates files were prepared, considering a discrete Poisson-based model. The focus was to detect areas of high infection rate. A criterion of ‘no geographical overlap’ was used to report secondary clusters. The P-value was generated using a combination of standard Monte Carlo, sequential Monte Carlo and Gumbel approximation [15]. We used 9999 Monte Carlo replications. The maximum temporal cluster size was set at 50%. The spatial window shape was circular, and the maximum spatial cluster size was set at 25% of the population at risk, after evaluating the effects of switching this value to 15%, 35% and 50%. Given that we used the geographic coordinates of the households, which were close to one another, we performed the analysis by evaluating the possible changes while using different levels of maximum spatial cluster size restriction as a percentage of the population at risk. We started arbitrarily with 50%, then 35%, 25% and finally 15%. This helped to show how the SatScan software captures the clustering with varying spatial cluster size restrictions. We observed that the centres of the circles of the most likely clusters of all spatial cluster size restrictions occurred consistently very close to the south-east corner of the village.

A maximum spatial cluster size of 50% is recommended because it should capture all clustering. In our case, it captured all the smaller clusters (most likely and secondary) that we observed while running the analysis with smaller maximum spatial cluster size within one most likely cluster, and it provided no secondary cluster. The greater portion of the cluster circle had no households in it. The clusters with maximum cluster size smaller than 50% yielded smaller clusters with varying relative risks. A maximum spatial cluster size of 35% yielded a very small secondary cluster with only 34 people in it, and the greater portion of the cluster circle did not hold households. A maximum spatial cluster size of 15%, to accommodate an additional secondary cluster, pushed the most likely cluster to the edge of the village and again the greater portion of the cluster circle contained no households. This revealed three secondary clusters, of which one contained only 21 people and the other was the same as the secondary cluster detected using the 25% maximum spatial cluster size restriction. Therefore, we decided that the 25% maximum cluster size restriction was most appropriate to show the malaria clustering activity of the study area because the portion of the most likely cluster circle with no households was relatively small and this specification yielded a secondary cluster. All the results reported here were obtained with this maximum spatial window size. The same maximum spatial cluster size was used to investigate the differences in clustering by the type of malaria and years of study. (Supplemental figures S1, S2 and S3 are provided showing space-time clusters of different maximum spatial cluster size restrictions.).

#### Individual level factors

The PASW Statistics 18 program (Chicago, IL, USA) was used to fit a generalized Poisson loglinear model. The dependent variables were episodes of vivax and falciparum malaria. The potential predictors considered were: distance to the vector breeding site, number of households located between each household and the vector breeding site (household count), sex, age, wealth index and total number of nights spent under insecticide-treated nets (ITNs). Every week, residents were asked whether they slept under ITNs the night before the interview and the names of the household member who had slept under ITNs were recorded. To get the total number of nights spent under ITNs, we summed-up the weekly data for each individual. The number of weeks for which each individual had been observed was used as a scale weight variable. The scale parameter method was Pearson chi-square and a robust estimator was used for the covariance matrix. The log-likelihood function was kernel. The ratio of the Pearson chi-square value to its degrees of freedom was used to rule out over-dispersion. This value was 1.34 and 1.28 for vivax and falciparum episodes, respectively. Given that these values did not deviate significantly from 1, we assumed that the Poisson distribution was a good fit for the data. Statistically significant (P-value <0.05) variables detected during bivariate analyses were considered for the multivariate model. The exponential form of the estimates was interpreted as the incidence rate ratio (IRR). We reported the IRR with 95% confidence intervals (CI).

We used ESRI ®ArcMap™ 9.3(CA, USA) to calculate the distance of each household from the vector breeding site (in km) and to produce the maps.

To get household count**–** the number of households between an individual household and the breeding site, we used the methodology described in supplemental file S1 (with figures S4, S5 and S6). This methodology assumes the potential possible area covered by a mosquito increases with distance travelled, and the flight pattern has a form of a circular sector. The measure is expressed in form of an angle. To highlight the sensitivity to the selection of search angle, the analysis was repeated with angles from 1 to 30 degrees (1, 5, 10, 15, 20, 25 and 30). The algorithm was implemented in R [16], and the household count for each search angle was analysed independently.

A recent paper by the authors has reported the predictors of falciparum malaria episodes, which included the effect of meteorological covariates (total rainfall, temperature and relative humidity), ITN utilization rate, efficacy of the insecticides used for IRS, and other factors. The statistical models employed were auto-regressive integrated moving average models with a transfer function model, generalized Poisson loglinear model and generalized estimating equation with logit link function. Principal Component Analysis was used to construct wealth index. The variables included were presence of electricity, watch, radio, TV, mobile telephone, refrigerator, separate room used for kitchen, bicycle, any land used for agriculture, livestock, account in bank or credit association and latrine facility. In addition the main materials of the floor, the roof and the wall were included. The technical details of the construction of the wealth index were reported elsewhere [17].

### Ethical Approval

The Regional Health Research Ethics Review Committee of the Southern Nations, Nationalities and People’s Regional Health Bureau has approved this research project. Informed verbal consent was obtained from all study participants and recorded by the research team on the ethical consent form (with prior approval from our ethics review committee). For minors, consent was obtained from their caregivers or legal guardians. All cases of malaria were treated immediately. Given that the blood samples were collected only for the purpose of malaria diagnosis and treatment, written consent was not suggested by the Regional Health Research Ethics Review Committee.

## Results

The study population (average of the three censuses) was 6631 in 1388 households (1346 locations). Within the study period, the total number of both types (*Plasmodium falciparum* and *vivax*) of malaria episode was 622, resulting in 45.1 episodes per 1000 persons per year. The number of *Plasmodium falciparum* and *vivax* episodes was 316 (in 226 locations) and 306 (in 199 locations); meanwhile, the annual rate of episodes per 1000 persons was 22.9 and 22.2, respectively. A higher number of malaria episodes occurred in males and in individuals aged 5–14 years. The mean distance of households with malaria episodes from the vector breeding site was lower (Table 1).

**Table 1:** Malaria episodes by *Plasmodium* species, sex, age, number of locations and mean distance of locations (with and without episodes) from the vector breeding site.

			Types of malaria			
			*Plasmodium Falciparum*		*Plasmodium Vivax*	
			Number	Percent	Number	Percent
Number of episodes	Sex	Male	208	65.8	196	64.1
		Female	108	34.2	110	35.9
	Age in years	<5	45	14.2	71	23.2
		5–14	146	46.2	130	42.5
		15–24	85	26.9	71	23.2
		>24	40	12.7	34	11.1
	Total		316		306	
Annual number of episodes per 1000			22.9		22.2	
Number of locations			226		199	
Mean (SD) distance of locations fromthe vector breeding site (km)		With episodes	2.28 (0.36)		2.36 (0.34)	
		Without episodes	2.53 (0.33)§		2.51 (0.34)‡	

§ Number of locations: 1120.

‡ Number of locations: 1147.

SD: standard deviation.

### Space–time Analysis

**Figure 2:**
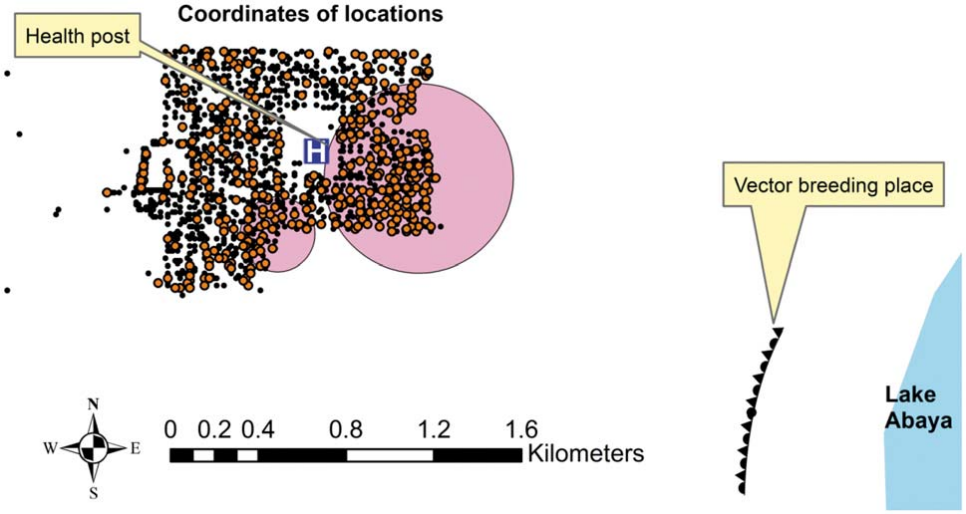
Most likely (big) and secondary (small) clusters (shaded circles) of all malaria episodes- orange dots refer to all malaria episodes.

**Figure 3:**
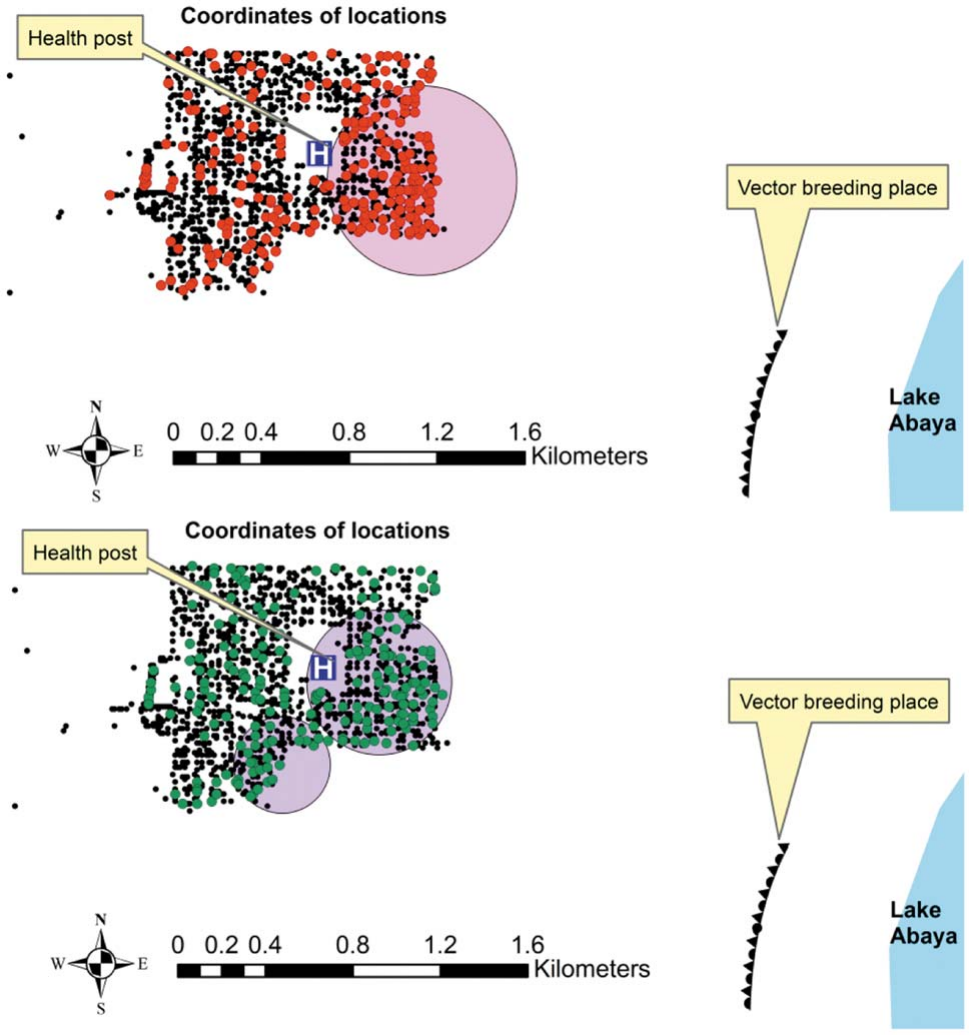
Most likely and secondary clusters (shaded circles) of malaria episodes by *Plasmodium* species: Red dots refer to *Plasmodium Falciparum* episodes that had one most likely cluster. Meanwhile, green dots refer to *Plasmodium Vivax* episodes that had most likely (big) and secondary (small) clusters.

Both the most likely and the secondary clusters were found on the south-east edge of the village, facing the vector breeding site (Figures 2 and 3).

#### The most likely cluster

The most likely space–time cluster lasted for 9 months (of the 25 months of the study) for both *Plasmodium* species. However, the *Plasmodium vivax* cluster started and ended 1 month earlier and had a smaller relative risk. The size and location of the most likely cluster of *Plasmodium falciparum* was the same as the cluster for both types of malaria. However, the relative risk for the *Plasmodium falciparum* cluster was greater. Households within the cluster were 7.49 times more at risk of contracting *Plasmodium falciparum* infection than households outside the cluster. This risk was 5.39 for *Plasmodium vivax* and 6.05 for both types of malaria (Table 2).

**Table 2:** Space**–**time scan statistics of the most likely cluster of malaria episodes.

	Both types of malaria	*Plasmodium Falciparum*	*Plasmodium Vivax*
Number of locations included	326	326	332
Coordinates	6.1100N, 37.6005E	6.1100N, 37.6005E	6.1105N, 37.5986E
Radius (km)	0.43	0.43	0.33
Time frame	Dec. 2009 to Aug. 2010	Dec. 2009 to Aug. 2010	Nov. 2009 to Jul. 2010
Population	1626	1626	1653
Number of episodes	230	133	106
Expected episodes	54.99	27.94	27.4
Annual episodes/1000	188.6	109.0	85.8
Observed/expected	4.18	4.76	3.87
Relative risk	6.05	7.49	5.39
Log likelihood ratio	184.43	124.51	77.11
P-value	<0.001	<0.001	<0.001

#### The secondary cluster

*Plasmodium falciparum* alone did not have a secondary cluster. The relative risk was 3.28 for both types of malaria and 5.47 for *Plasmodium vivax* alone. The time-span for both types of malaria was the same as that of the most likely cluster but was shorter by 4 months for *Plasmodium vivax* alone (Table 3).

**Table 3:** Space**–**time scan statistics of the secondary clusters.

	Both types of malaria	*Plasmodium Vivax*
Number of locations included	81	102
Coordinates	6.1077N, 37.5947E	6.1071N, 37.5946E
Radius (km)	0.17	0.22
Time frame	Dec. 2009 to Aug. 2010	Dec. 2009 to Apr. 2010
Population	407	489
Number of episodes	43	23
Expected episodes	13.76	4.48
Annual episodes/1000	140.8	113.8
Observed/expected	3.12	5.13
Relative risk	3.28	5.47
Log likelihood ratio	20.46	19.67
P-value	<0.001	<0.001

#### Analysis for separate years

**Figure 4:**
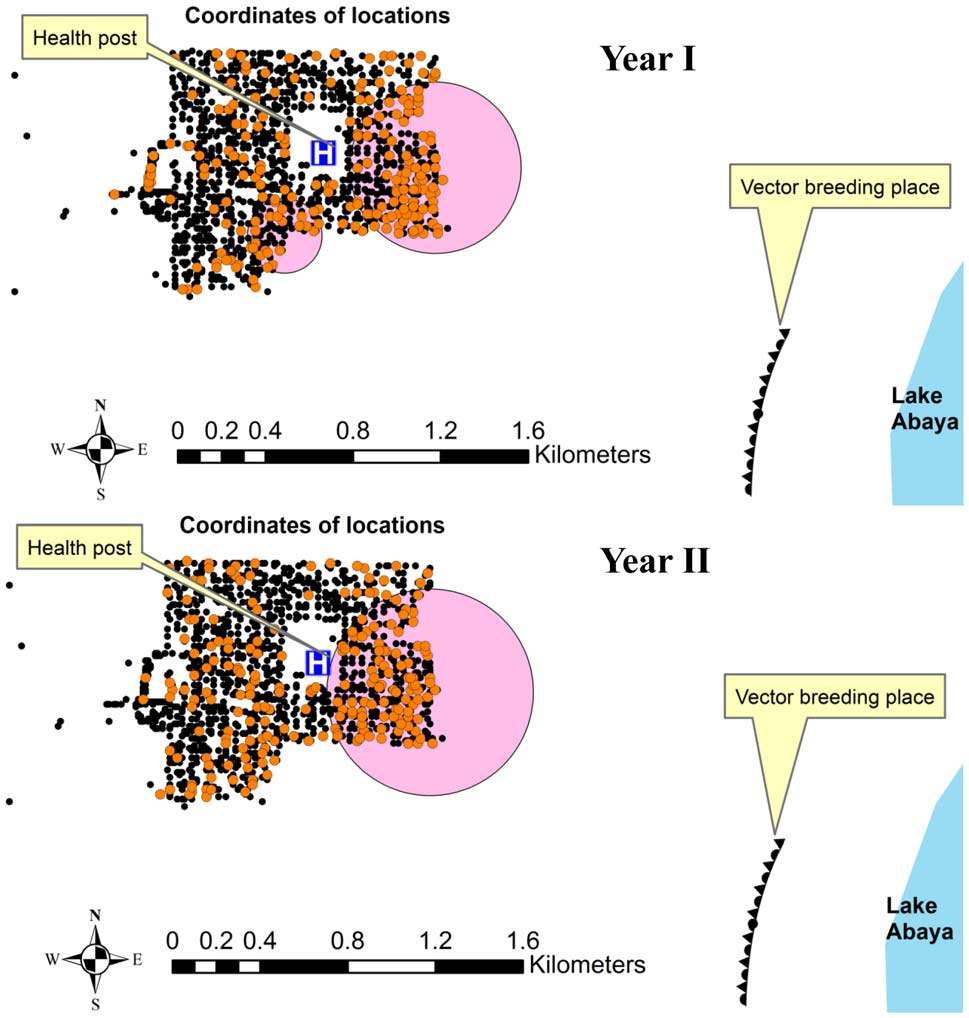
Most likely (big) and secondary (small) clusters (shaded circles) of malaria episodes (both types) according to study year- orange dots refer to all malaria episodes of the corresponding year.

The space**–**time analysis result of both *Plasmodium* species (for separate years) was relatively similar with the analysis involving the whole study period. The location of the most likely clusters of year I and year II was almost the same. However, significant secondary cluster was found only in the first year of the study. There was also slight increase in the relative risk values (Table 4). Figure 4 shows significant space**–**time clusters of each study year.

**Table 4:** Space**–**time scan statistics of the most likely and secondary clusters of malaria episodes (both types) according to year of study.

	Year I(Apr. 2009 to Mar.2010)		Year II(Apr. 2010 to Apr.2011)
	Most-likely cluster	Secondary cluster	Most-likely cluster
Number of locations included	264	79	322
Coordinates	6.1105N, 37.6009E	6.1077N, 37.5947E	6.1099N, 37.6009E
Radius (km)	0.39	0.17	0.47
Time frame	Dec. 2009 to Mar. 2010	Jan. 2010 to Mar. 2010	Apr. 2010 to Aug. 2010
Population	1335	399	1604
Number of episodes	107	22	115
Expected episodes	21.9	4.9	27.6
Annual episodes/1000	241.9	223.8	171.2
Observed/expected	4.89	4.52	4.2
Relative risk	6.77	4.77	6.21
Log likelihood ratio	97.79	16.52	93.13
P-value	<0.001	0.002	<0.001

### Malaria Preventive Measures and Spatio-temporal Clustering

**Figure 5:**
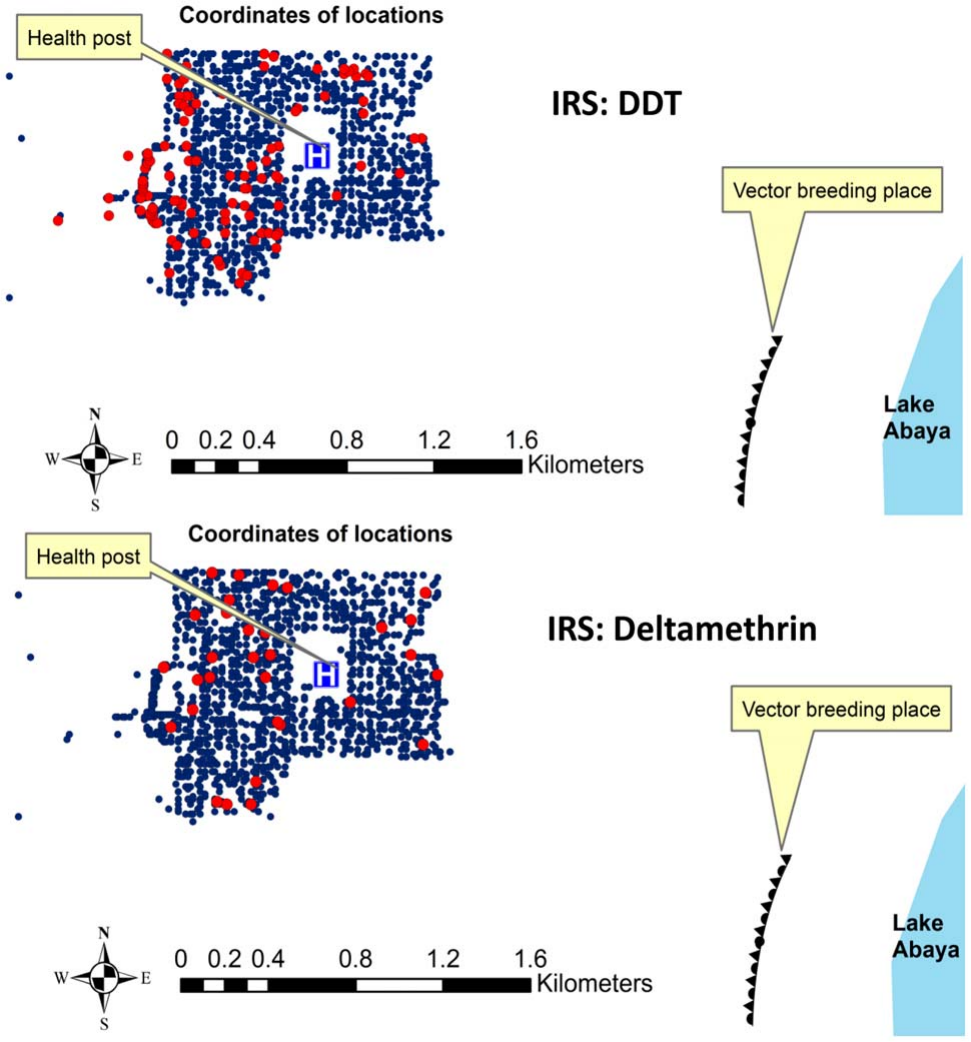
Spatial coverage of indoor residual spraying (IRS) with DDT and Deltamethrin: Red dots refer to houses that were not sprayed and smaller blue dots refer to sprayed houses.

**Figure 6:**
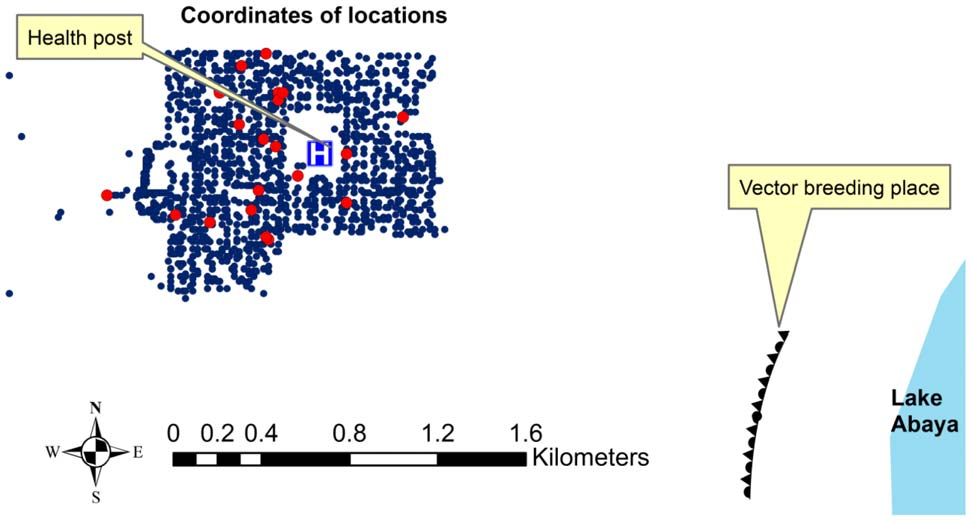
Spatial coverage of insecticide treated bednets (ITNs): Red dots refer to households that did not receive ITNs and smaller blue dots refer to those who received at least one ITN.

**Figure 7:**
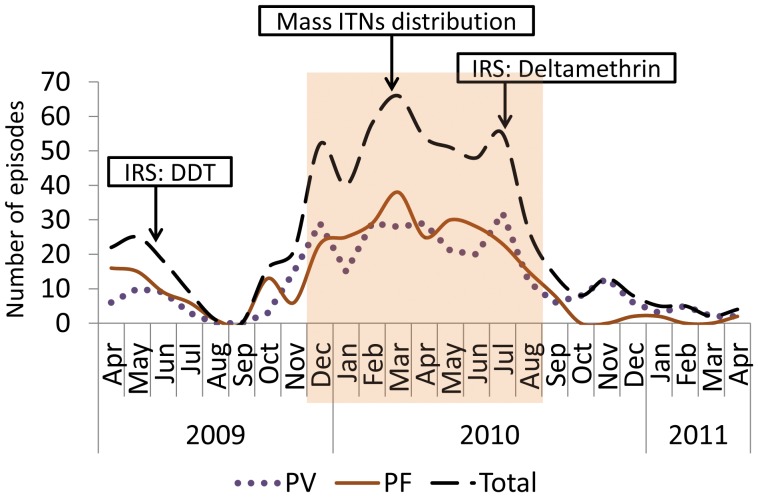
Monthly malaria incidence, time of interventions and time span of the most likely spatio–temporal cluster of all types of malaria. The shaded part indicates the time-span of the most likely space–time cluster.

The three major preventive measures applied by the government during the study period included: IRS with Dichlorodiphenyltrichloroethane (DDT): 91% of the houses were sprayed in June 2009, free mass ITN distribution (2.3 ITNs per household) in March 2010, and IRS with Deltamethrin: 97.5% of the houses were sprayed in July 2010. The spatial coverage of IRS and ITNs are presented in Figures 5 and 6. The spatio-temporal clustering started 5–6 months after the IRS with DDT and continued for 4–5 months after free mass distribution of ITNs, but ended within 1 month after IRS with Deltamethrin. The time-span of the most likely space–time cluster generated by SatScan and the timing of the interventions in relation to the monthly incidence of malaria are presented in Figure 7.

### Factors Determining Episodes of Falciparum and Vivax Malaria at Individual Level

Of the total number of falciparum and vivax episodes, 224 and 203 episodes, respectively, occurred among permanent residents for whom we had follow-up data.

Living nearer to the vector breeding site increased the risk of acquiring falciparum malaria, that is, each 1 km closer to the vector breeding site added 4.93 (95% CI: 2.59–9.35) times more risk. Household count was negatively associated with both types of malaria episodes with all vector’s search angles considered during bivariate analyses. However, the household counts of the first three lower search angles (1, 5 and 10 degrees) were found to be statistically significant in the multivariate model for falciparum episodes. As the vector’s search angle decreases, the effect of household count increases. With a search angle of 1 degree, for each additional household between a household of interest and the vector breeding site, the risk of getting falciparum malaria decreases by 2%. Male participants had 1.63 (95% CI: 1.22–2.18) times more risk of acquiring falciparum malaria. When compared with adults aged >24 years, children aged 5–14 years had 3.82 (95% CI: 2.52–5.78) times more risk. Having higher wealth index was marginally failed to be protective against falciparum malaria (P-value: 0.051) in the model containing household count. Nevertheless, for search angles above 15 degrees, the wealth index regains statistical significance as the household count becomes no more statistically significant. The total number of nights spent under ITNs was not associated with the total number of falciparum episodes. Regarding vivax malaria, household count, sex, wealth index and the total number of nights spent under ITNs were not significant predictors. Meanwhile, when compared with adults aged >24 years, children <5 years old had 7.6 (95% CI: 4.2–13.74) times more risk, and living 1 km closer to the vector breeding site conferred 2.9 (95% CI: 1.2–6.99) times more risk of acquiring vivax malaria (Table 5).

**Table 5:** Factors determining malaria episodes at individual level.

Variable (n = 8121)		*Plasmodium Falciparum* (224 episodes)		*Plasmodium Vivax* (203 episodes)		
		Crude IRR (95% CI)	Adjusted IRR (95% CI)	Crude IRR (95% CI)	Adjusted IRR (95% CI)	
Distance (km) fromvector breeding site§		11.11(6.67–20.0)	4.93(2.59–9.35)*	4.55(2.7–7.69)		2.9(1.2–6.99)*
Household count with a searchangle of 1 degree‡		0.98(0.96–0.99)	0.98(0.96–0.99)*	0.97(0.96–0.98)		0.99(0.97–1.01)
Sex : Male		1.65(1.23–2.23)	1.63(1.22–2.18)*	1.26(0.87–1.83)		NA
Age in years‡	<5	3.37(2.02–5.62)	3.07(1.87–5.03)*	7.82(4.31–14.2)		7.6(4.2–13.74)*
	5–14	4.32(2.83–6.58)	3.82(2.52–5.78)*	6.91(3.95–12.12)		6.53(3.74–11.41)*
	15–24	2.1(1.28–3.44)	2.14(1.31–3.48)*	1.84(0.91–3.74)		1.84(0.91–3.75)
Wealth index		0.76(0.65–0.89)	0.91(0.83–1.0)¥	0.95(0.78–1.16)		NA
Total number of nightsspent under ITNs		1(0.99–1.01)	NA	1(0.99–1.01)		NA

‡ Reference category: >24 years.

* Significant at P-value <0.05.

§ The reciprocal of the IRR (95% CI) was presented to show the risk of being closer to the vector breeding site.

‡ Household count refers to the number of households located between each household and the vector breeding site. For search angles of 5 and 10 degrees, the effect measures, adjusted IRR (95% CI), became 0.995 (0.991–0.999) and 0.997 (0.995–0.999), respectively.

¥ P-value: 0.051.

NA: Not applicable.

## Discussion

There was a space–time clustering of malaria at household level. Free mass distribution of ITNs did not affect the spatio-temporal clustering of malaria, but IRS might have. Living nearer to the vector breeding site increased the risk of acquiring malaria infection. These differences in malaria risk within a population who live in one village reflect the complexity of the disease transmission dynamics.

Our study confirms the findings of other studies that have shown spatio-temporal clustering of malaria cases at varying geographical extents [9]–[13], and even at household level [8], [14]. The epidemiology of malaria in the study area may be unique, and this will limit the generalizability of the findings. However, the study of malaria in such a micro-environment may help to provide better knowledge on how the disease transmission is influenced by preventive and control measures.

Clustering varied by the type of malaria [8]. The similarity in size and location of the most likely clusters for both types and for *Plasmodium falciparum* malaria may show that the clustering of *Plasmodium falciparum* dominated that of *Plasmodium vivax*. It was also shown that the relative risk of the *Plasmodium falciparum* cluster was higher than that of both types of malaria for the same cluster location and size. Similarly, the existence of a secondary cluster in the analysis of both types of malaria took into account the clustering activity of *Plasmodium vivax* (rather than *Plasmodium falciparum*) because we observed a secondary cluster only for this species (with a shorter time span and higher relative risk) when analysed separately. The most likely cluster of *Plasmodium vivax* alone was also closer to the centre of the village (farther from the vector breeding site). Descriptive statistics also showed that the mean distance of households with episodes of *Plasmodium vivax* was slightly farther from the vector breeding site than that of households with *Plasmodium falciparum* episodes. To confirm the effect of the vector breeding site on malaria clustering in households close to it, we used Poisson regression to estimate its effect at the level of individual episodes, controlling for household count, sex, age, wealth index and the number of nights slept under ITNs. Those who lived nearer to the vector breeding site had a greater risk of acquiring infection; meanwhile, the increase in risk was greater for falciparum than for vivax malaria (4.93 versus 2.9), respectively, as a function of living 1 km closer to the vector breeding site. This may support the finding that the most likely cluster of falciparum malaria was nearer to the vector breeding site than the most likely cluster of vivax malaria, and also justifies the need for separate space–time clustering analysis for each *Plasmodium* species. There may also be a need to verify the role of new versus relapse episodes of vivax malaria on the space–time clustering.

An increase in risk of getting vivax malaria has occurred in two locations (vivax malaria had significant secondary cluster) and the relative risks within vivax malaria clusters were smaller than that of falciparum malaria cluster. This may suggest that a targeted intervention could be easier to apply for falciparum than for vivax malaria in the study area.

There is ample evidence that sleeping under ITNs protects against malaria infection [18], provided that they are used properly [19]. The absence of a significant impact of the number of nights slept under ITNs on the total number of malaria episodes an individual experienced may be related to inconsistent or improper use of ITNs. Meanwhile, in the prevention of malaria at a community level, the role of ITNs depends on the utilization rate [20]. The level of ITN utilization increased after mass distribution of ITNs (data are not shown); however it did not lower the risk for malaria clustering. Therefore, it is possible that free mass distribution of ITNs is not an effective tool with which to combat malaria without follow-up to ensure the optimal utilization of the ITNs.

The time span (December 2009 to August 2010) for the spatio-temporal clustering of both types of malaria ended when the possible effect of IRS with Deltamethrin (sprayed in July 2010) started. Although it may not be possible to reach the conclusion that IRS alone eliminated the spatio-temporal clustering of malaria without considering the effects of other factors such as rainfall and temperature, it is also not possible to state that the timing of the possible effect of IRS and the end of the clustering coincided simply by chance. Thus, we suggest that IRS with Deltamethrin has possibly suppressed the transmission to the level where little power to identify clusters remains. Meanwhile, the location where we observed the clustering activities was almost perfectly covered with IRS where only fewer than 10 households did not receive the intervention. A recent paper by the authors has discussed the reasons for the differences in the risk of falciparum malaria with regard to sex, age, wealth index, ITN use (with a 2-week lag in the effect) and other factors. We also showed that, among the meteorological covariates, rainfall (with a lag of 6 weeks) was a significant predictor of falciparum malaria. When controlled for the effect of rainfall, IRS with Deltamethrin significantly reduced the incidence of falciparum malaria; however, utilization rate of ITNs did not [17]. This finding is consistent with the negative effect of IRS with Deltamethrin and the ‘null’ effect of mass distribution of ITNs on the clustering of malaria episodes presented here.

All the clusters observed were on the south-east side of the community, and near to the identified vector breeding site on the shore of Lake Abaya. This implies that the greater risk of malaria infection among these households served as a ‘barrier’ between the breeding site and households that lived to the north-west of the cluster. This was supported by the analysis showing the significant effect of number of households located between each household and the vector breeding site while using the vector’s search angle scenarios of 1, 5 and 10 degrees. Meanwhile, the clustering activity observed close to the vector breeding site in our study area could provide an example of what Bousema et al. described as “hotspots of malaria transmission in the dry and wet season”. These hotspots were referred to as groups of households that have an increased risk of malaria infection within a focus of malaria transmission [21], and cognizant of the need for focused intervention, studies suggested ways of identifying malaria hotspots [22], [23].

The edge effect is worth mentioning, because we observed clustering activity on the edge of the village. An edge effect may result in a biased estimate of risk at the edge of a study area provided that there are no data for the adjacent localities [24]–[26]. In this study, beyond the edge of the study area in which we observed the clustering activity, there were no residential houses or populations at risk – farmland extended to the vector breeding site. Therefore, we presume that the relative risk reported here is unlikely to have been influenced by an edge effect [26], [27]. Qualitative evaluation of the figures also confirms that malaria episodes were more concentrated in the location where the SatScan identified the clustering. In addition, we chose a spatial window size that caused the greater portion of the cluster circle to move inwards [28].

### Conclusions

The risk of malaria infection varied within one village, and there was spatio-temporal clustering of malaria episodes at household level. The vector breeding site identified may have played a role in the clustering of malaria. Mass distribution of ITNs did not influence the spatio-temporal clustering of malaria, but IRS with Deltamethrin might have eliminated the clustering activity. Local knowledge of malaria transmission and follow-up on ITN use, combined with targeted interventions, may improve the existing malaria prevention and control efforts.

## Supporting Information

Figure S1 **Space–time clusters of maximum spatial cluster size restriction of 15 per cent.**
(TIF)

Figure S2 **Space–time clusters of maximum spatial cluster size restriction of 35 per cent.**
(TIF)

Figure S3 **Space–time clusters of maximum spatial cluster size restriction of 35 per cent.** Figures S1, S2 and S3 show how the different maximum spatial cluster size restrictions given to the SatScan affect quantitative and qualitative outcomes. The largest/larger circle in each figure represents the most likely cluster, meanwhile, the smaller circles/circle represent/s significant secondary clusters. The third secondary cluster in Figure S1 is indicated by a pink dot to the south of the health post. This cluster is the smallest with a radius of 11 meters and composed of 21 people in three households. The relative risk (14.19) of this cluster is the highest of all clusters so far presented.
(TIF)

Figure S4 **Number of households (59) between the breeding site (B) and a household (H) using the simplistic (rectangular) approach.**
(TIF)

Figure S5 **Illustration of the parameters used to estimate the number of households between a breeding site (B), and a household (H), separated by a distance (D), and a mosquito flying with a search angle (A).**
(TIF)

Figure S6 **Taking the approach of a search angle rather than a constant search width alter the perception of how many houses a mosquito potentially must pass to reach a given house.** This figure is showing three search angles: 10° (567 houses), 5° (284 houses), and 1° (55 houses).
(TIF)

File S1 **Description of methodology employed to get number of households located between each household and the vector breeding site.**
(DOCX)
