# Supplementary figures and images for: Effect of Bednets and Indoor Residual Spraying on Spatio-Temporal Clustering of Malaria in a Village in South Ethiopia: A Longitudinal Study
Source: PLoS One. 2012 Oct 12;7(10):e47354. doi: 10.1371/journal.pone.0047354 (PMC3470588; doi:10.1371/journal.pone.0047354)

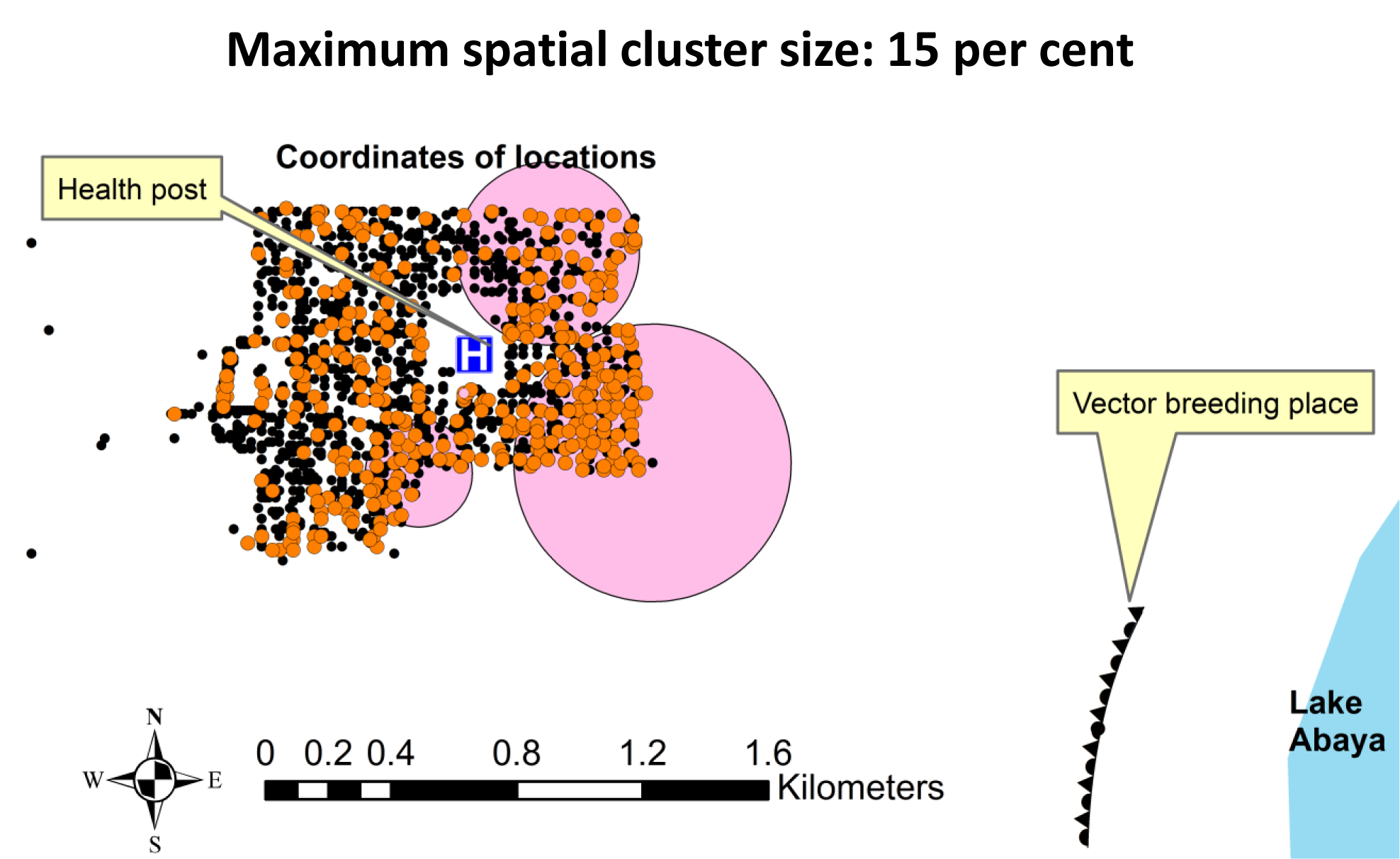

Supplement: Figure S1 — Space–time clusters of maximum spatial cluster size restriction of 15 per cent. (TIF) [file pone.0047354.s001.tif]

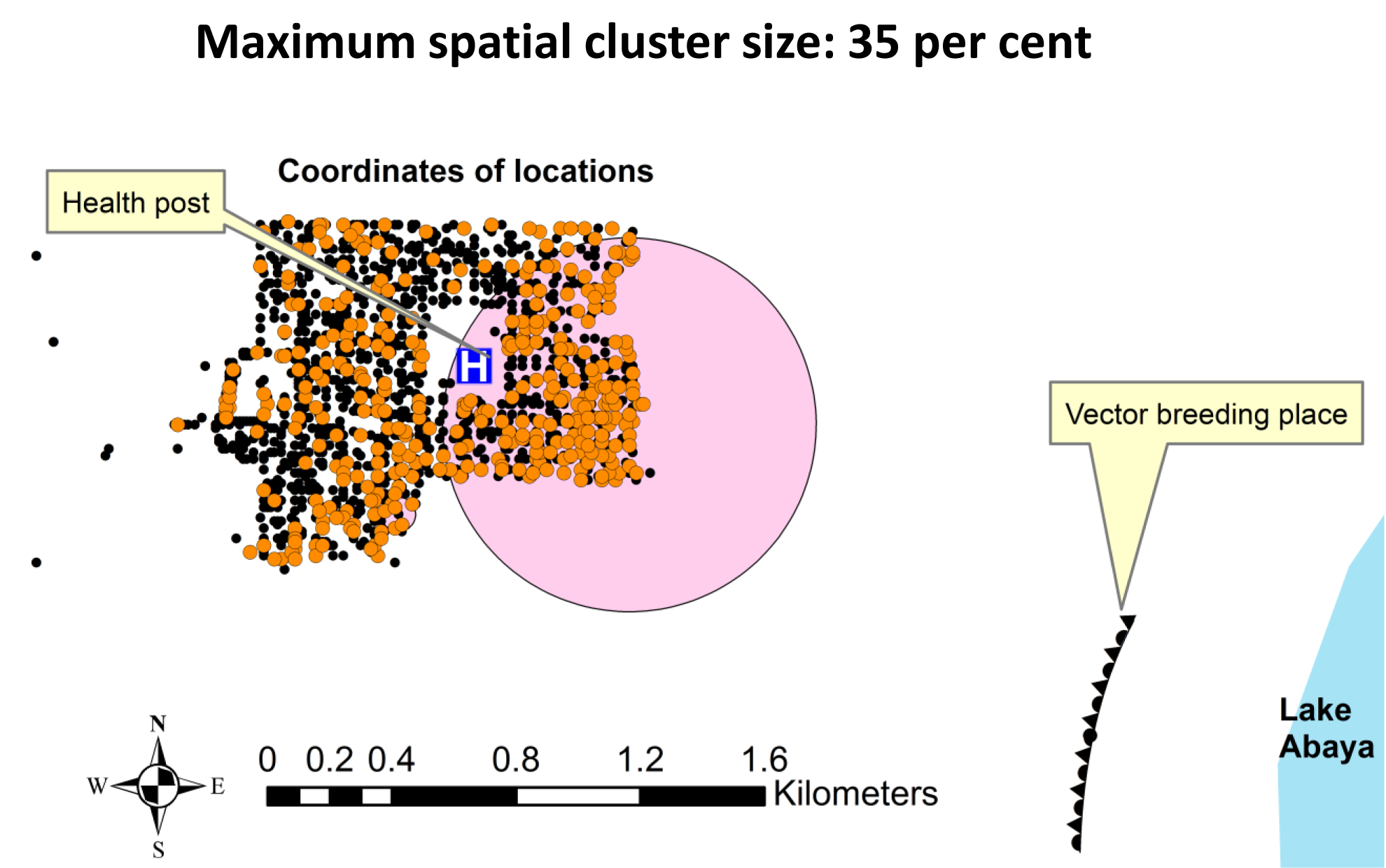

Supplement: Figure S2 — Space–time clusters of maximum spatial cluster size restriction of 35 per cent. (TIF) [file pone.0047354.s002.tif]

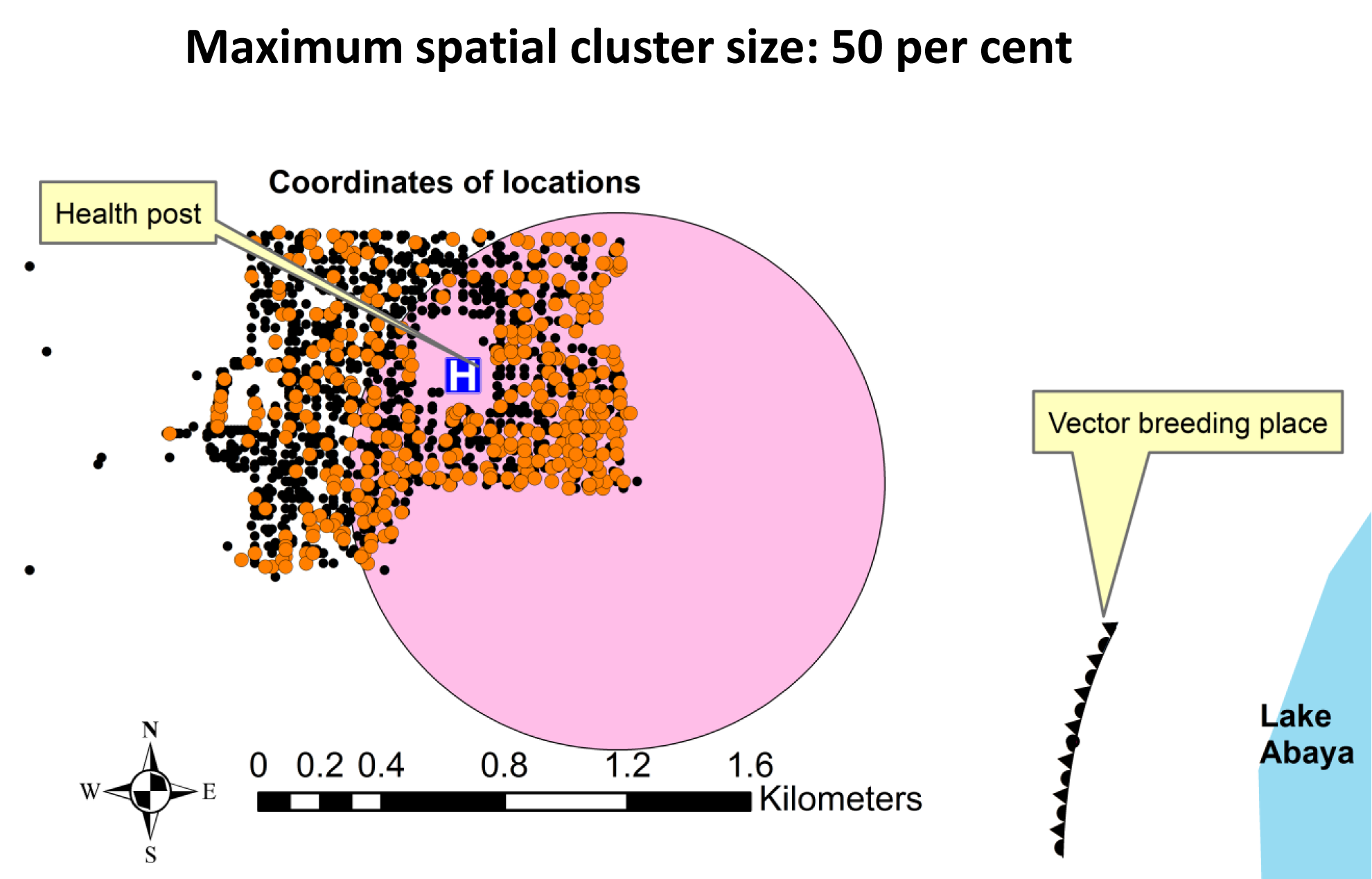

Supplement: Figure S3 — Space–time clusters of maximum spatial cluster size restriction of 35 per cent. Figures S1, S2 and S3 show how the different maximum spatial cluster size restrictions given to the SatScan affect quantitative and qualitative outcomes. The largest/larger circle in each figure represents the most likely cluster, meanwhile, the smaller circles/circle represent/s significant secondary clusters. The third secondary cluster in Figure S1 is indicated by a pink dot to the south of the health post. This cluster is the smallest with a radius of 11 meters and composed of 21 people in three households. The relative risk (14.19) of this cluster is the highest of all clusters so far presented. (TIF) [file pone.0047354.s003.tif]

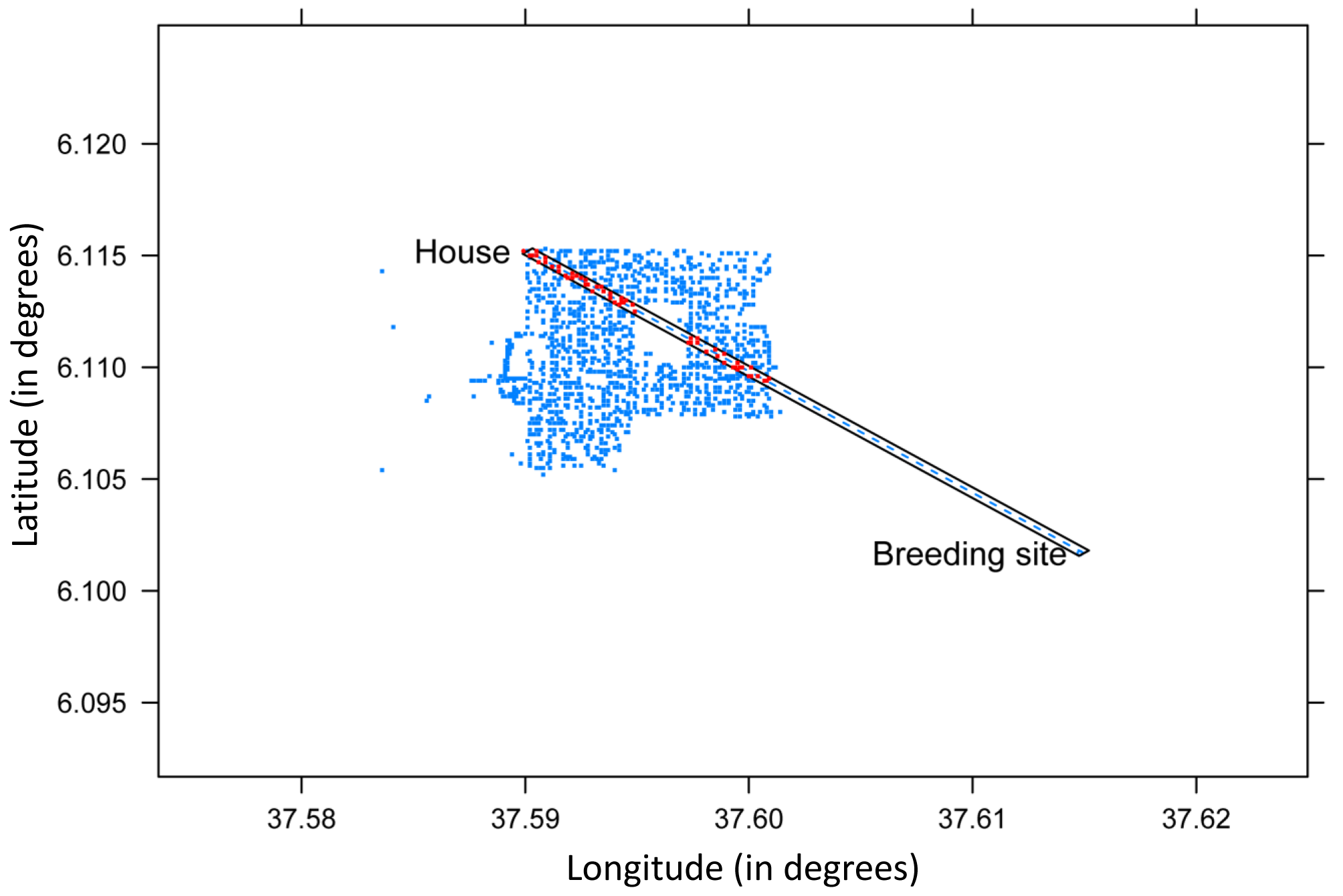

Supplement: Figure S4 — Number of households (59) between the breeding site (B) and a household (H) using the simplistic (rectangular) approach. (TIF) [file pone.0047354.s004.tif]

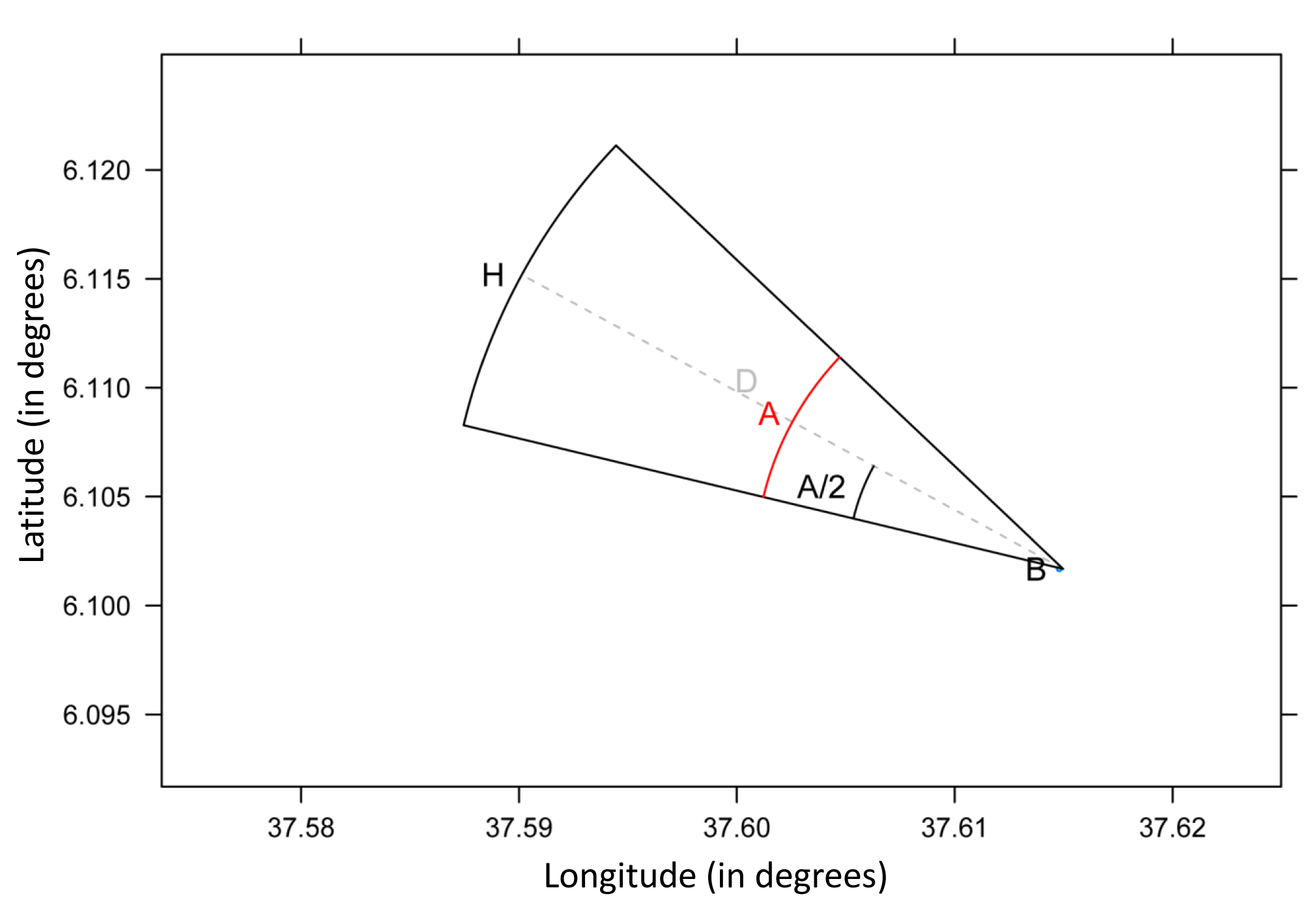

Supplement: Figure S5 — Illustration of the parameters used to estimate the number of households between a breeding site (B), and a household (H), separated by a distance (D), and a mosquito flying with a search angle (A). (TIF) [file pone.0047354.s005.tif]

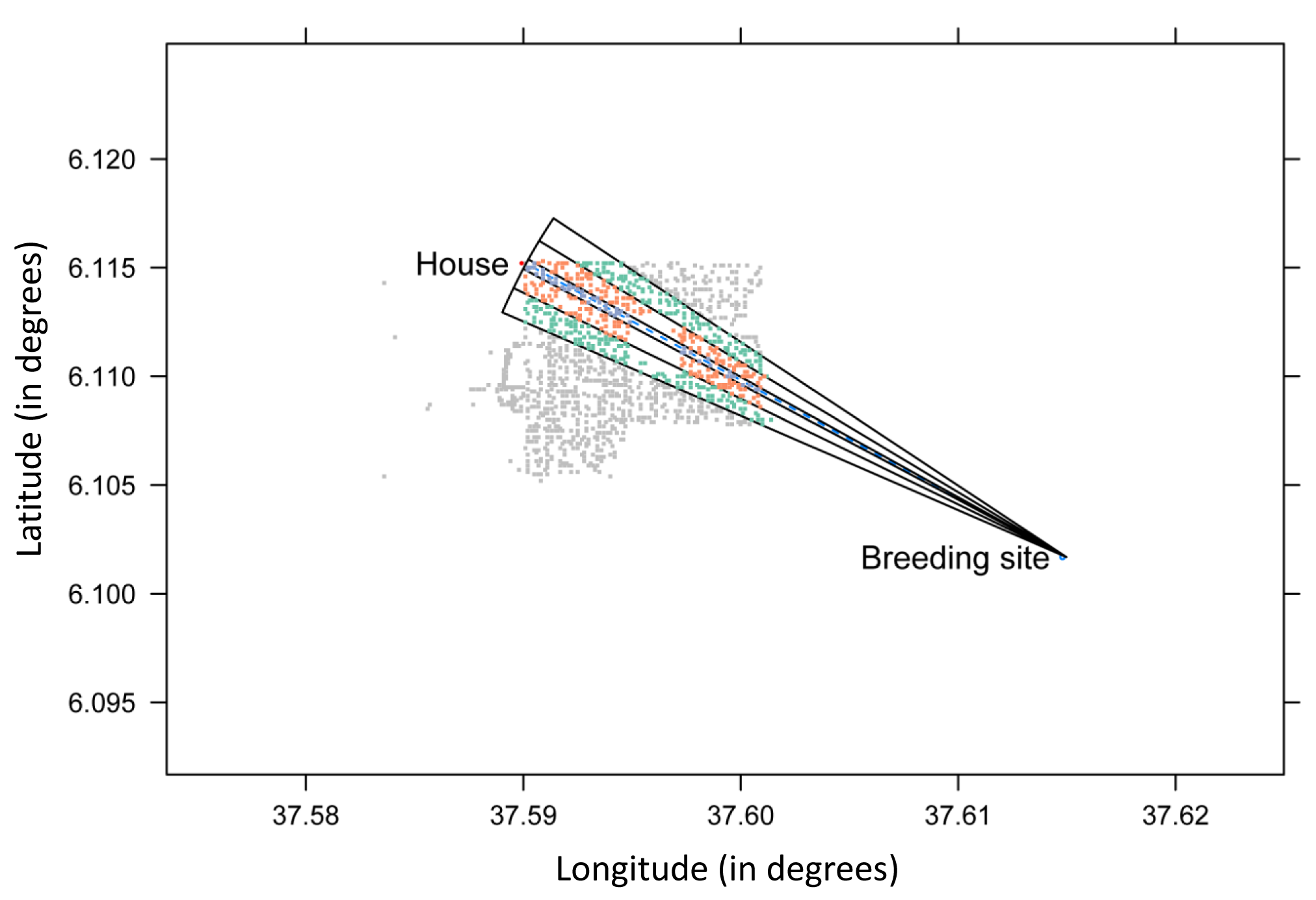

Supplement: Figure S6 — Taking the approach of a search angle rather than a constant search width alter the perception of how many houses a mosquito potentially must pass to reach a given house. This figure is showing three search angles: 10° (567 houses), 5° (284 houses), and 1° (55 houses). (TIF) [file pone.0047354.s006.tif]
