# Supplementary material for: Effect of Bednets and Indoor Residual Spraying on Spatio-Temporal Clustering of Malaria in a Village in South Ethiopia: A Longitudinal Study
Source: PLoS One. 2012 Oct 12;7(10):e47354. doi: 10.1371/journal.pone.0047354 (PMC3470588; doi:10.1371/journal.pone.0047354)
Supplement: File S1 — Description of methodology employed to get number of households located between each household and the vector breeding site. (DOCX) [file pone.0047354.s007.docx]

**Problem**

To make a clear distinction between distance from a breeding site and number of households between a household and the breeding site, some assumptions has to be made about mosquito behaviour and flight patterns. In cases where the terrain is relatively flat, the distance between a breeding site (B) and a household (H) can easily be found using for example Great Circle distance. The counting of number of households can take many forms, and here we show some examples on how this can be calculated.

**Methods**

**Simplistic approach**

Let us consider an anopheline breeding site (B) and a household (H). If we assume a mosquito (M) is heading towards the given household, the most simplistic approach would assume the mosquito is flying in a straight line, from B to H, and that the deviation from the line is independent of the distance flown. In this case we can assume flight is restricted inside a rectangular shape, and the number of households inside the rectangular polygon, minus one, is the number of households between B and H. Figure S4 shows an example of the houses which would be counted in case of the simplistic approach.

**More realistic approach**

To complicate, or maybe making the counting more realistic, we still assume that when leaving the breeding site, the mosquito is heading for the same household. This time, however, we assume the deviation from the line is dependent on the distance own. In this case, the deviation is measured as an angle, A, which is unknown since the true angle has not been measured in the field.

The polygon (a circular sector) can be constructed using an iterative procedure defining the polygon nodes (in addition to the breeding site which is the start and end node). D is equal to the distance from the household (H) to the breeding site (B) in meters. An illustration of the method can be seen in figure S5.

For a search angle A (in deg), the angle at the nodes $\theta_{n}$is: $\theta_{n}=MOD\left(\text{atan}2\left(H_{lat}-B_{lat},H_{lon}-B_{lon}\right)+\frac{A_{n}\cdot pi}{180},pi\right)+\frac{pi}{2}$ (1)

$$\begin{matrix} \text{with }A_{n}=-A/2,...,A/2 \\ \text{ Then }\Delta X\text{ and }\Delta Y\text{ become} \end{matrix}$$

$\Delta X=D\cdot\cos\left(\theta_{n}\right)$ (2)

$\Delta Y=D\cdot\sin\left(\theta_{n}\right)$ (3)

$$\text{and the }\Delta lon\text{ and }\Delta lat\text{ (}lon=\text{longitude, }lat=\text{latitude) become}$$

$\Delta lon=\frac{\Delta X}{f_{x}\cdot\cos\left(H_{lat}\cdot\frac{pi}{180}\right)}$ (4)

$\Delta lat=\frac{\Delta Y}{f_{y}}$ (5)

$$\text{Where }f_{x}\text{ and }f_{y}\text{ are flattening factors for the WGS84 ellipsoid}$$

$f_{x}=6378137\cdot\frac{pi}{180}$ (6)

$f_{y}=6356752.3142\cdot\frac{pi}{180}$ (7)

and the longitude of the node $\left(N\right)$is $N_{lon}=B_{lon}+\Delta lon$and the latitude is $N_{lat}=B_{lat}+\Delta lat$.

A point in polygon operation following Pebesma and Bivand [1] and Bivand et al. [2] is used to identify which houses are in the flight path of the mosquito.

Figure S6 shows an example of the consequences of changing the search angle with 10º (567 houses), 5º (284 houses), and 1º (55 houses).

**Discussion**

Here we briefly described two methods to calculate the number of houses between a breeding site, B, and a household, H. Since it is not known how the mosquitoes fly in order to reach a house, this document is focusing on the assumptions and methodologies made in order to calculate this index. To construct a robust index, high resolution release-recapture experiments must be carried out. Such experiments were not part of this work.

1. Pebesma EJ, Bivand RS (2005). Classes and methods for spatial data in R. R News 5 (2), <http://cran.r-project.org/doc/Rnews/>
2. Bivand RS, Pebesma EJ, Virgilio GR (2008). Applied spatial data analysis with R. Springer, NY. http://www.asdar-book.org/
